# Impact of Deoxynivalenol, Cytokines, and Particulate Matter on Human Trophoblast Cells

**DOI:** 10.3390/ijms27146434

**Published:** 2026-07-20

**Authors:** Deguang Liu, Johan Garssen, Betty C. A. M. van Esch, Astrid Hogenkamp

**Affiliations:** 1 Division of Pharmacology, Utrecht Institute for Pharmaceutical Sciences, Faculty of Science, Utrecht University, 3584 CG Utrecht, The Netherlands; d.liu2@uu.nl (D.L.); j.garssen@uu.nl (J.G.); e.c.a.m.vanesch@uu.nl (B.C.A.M.v.E.); 2 Danone Research & Innovation, 3584 CT Utrecht, The Netherlands

**Keywords:** placenta, deoxynivalenol, particulate matter, trophoblast, inflammation, interleukin-6, human chorionic gonadotropin

## Abstract

Placental inflammation and dysfunction play critical roles in adverse pregnancy outcomes, including preeclampsia and intrauterine growth restriction. Exposure to environmental factors, such as dietary toxins, infectious agents, and pollution, is thought to increase the risk of these outcomes. Despite the widespread use of HTR-8/SVneo and BeWo cells as trophoblast models, their comparative sensitivity to maternal exposome-related stimuli has not been systematically evaluated within the same experimental framework. we examined the responses of HTR-8/SVneo and BeWo trophoblast cell lines to mycotoxin deoxynivalenol (DON), an inflammatory cytokine cocktail (CC; TNF-α, IL-1β, IFN-γ), and particulate matter (PM2.5). Cells were seeded with either 0.2% or 1% penicillin/streptomycin (P/S) and exposed for 24 h. Cytokine secretion (IL-6 and IL-8), human chorionic gonadotropin (hCG) production, and mRNA and protein expression of junctional markers (ZO-1, OCLD, CLDN-3, CLDN-4, E-CAD) were analysed. DON significantly increased IL-6 secretion in both cell lines and elevated IL-8 levels in HTR-8/SVneo cells only. DON suppressed hCG production in both cell lines. CC exposure markedly elevated IL-6 and IL-8 levels, particularly in HTR-8/SVneo cells, without affecting hCG level. PM exposure did not significantly alter IL-6, IL-8, or hCG levels in either cell line. DON and CC altered the mRNA and protein expression of junctional markers in BeWo cells(ZO-1, OCLD, CLDN-3,CLDN-4), whereas HTR-8/SVneo cells showed more limited changes. Most cellular responses were consistent across both P/S concentrations. HTR-8/SVneo and BeWo cells exhibit differential sensitivity to DON, CC, and PM2.5, underscoring the importance of cell model selection in in vitro placental toxicology research. These results provide a comparative framework for interpreting trophoblast responses to maternal exposome-related stimuli and highlight the need for functional validation in future studies.

## 1. Introduction

The human placenta serves as the interface between maternal and fetal circulations, composed of various cell types including cytotrophoblasts (CTBs), syncytiotrophoblasts (SCTs), and extravillous trophoblasts (EVTs). CTBs differentiate into SCTs and EVTs, which play key roles in nutrient and gas exchange, hormone production, and maternal artery remodeling. These processes can be disrupted by external stimuli from the maternal environment, which may impair placental function. In the current study, we used two different placental cell lines to make a thorough comparison of their responses to different types of potentially detrimental stimuli [1,2]. While neither cell line fully replicates the in vivo functions of SCTs or EVTs, each offers unique advantages for studying different aspects of placental biology and function. Importantly, despite the widespread use of both cell lines in placental research, no study has directly and systematically compared their sensitivity to maternal exposome-related stimuli within the same experimental framework—a gap that limits interpretation of inter-study variability and informed model selection. In addition, because preliminary unpublished observations from our laboratory suggested that HTR-8/SVneo cells and BeWo cells may be less responsive to certain stimuli under standard antibiotic concentrations, we included two concentrations of penicillin/streptomycin (0.2% and 1% P/S) as a methodological validation step to assess whether antibiotic concentration influenced cellular responses to the selected stimuli.

For our experiments, we chose three different types of stimuli to serve as representatives of the putative detrimental components of the maternal exposome; (1) exposure to the mycotoxin deoxynivalenol (DON); (2) exposure to a cytokine cocktail composed of Tumor Necrosis Factor alpha (TNF-α), Interleukin-1 beta (IL-1β) and Interferon gamma (IFN-γ) and (3) exposure to particulate matter (PM).

First, DON is a mycotoxin produced by Fusarium fungi, commonly found in contaminated grains and cereals [3,4,5]. It has severe adverse effects on human health, including immunotoxicity and reproductive issues [5,6]. Pregnant women can be exposed to DON through food, which poses significant risks to maternal and fetal health [7,8,9]. DON can cross the placental barrier, potentially affecting fetal development [10]. Investigating the responses of BeWO and HTR-8/SVneo cells to DON may help understand its role in pregnancy complications, as EVTs are crucial for placental development and function.

Second, the effects of BeWo and HTR-8/SVneo cell exposure to a cytokine cocktail (CC) were studied as a proxy for maternal immune activation, given that TNF-α, IL-1β, and IFN-γ are cytokines released during inflammation. Since maternal immune activation is associated with adverse pregnancy outcomes, including preterm birth, low birth weight, and developmental disorders in the offspring [11,12,13,14], understanding the differential responses of trophoblast cells to inflammation may contribute to the development of targeted interventions aimed at mitigating these risks.

Exposure to PM2.5 was chosen as the third stimulus due to its common prevalence as an environmental pollutant, particularly in urban and industrial areas. PM2.5 can penetrate deep into the lungs and enter the bloodstream, leading to systemic inflammation and endocrine disruption [15,16]. The presence of PM in maternal and fetal circulation highlights the potential risks associated with direct fetal exposure [17]. Furthermore, black carbon particles, a component of PM2.5, have been found to accumulate in the placenta and cord blood, suggesting the potential for direct fetal exposure during pregnancy [18] and highlighting the need for more insight in the underlying mechanisms by which PM2.5 may contribute to potential detrimental long-term health effects. Understanding the responses of trophoblasts to maternal environmental triggers is crucial to develop interventions to mitigate the risks associated with exposure during pregnancy.

In this study, we systematically compared the responses of HTR-8/SVneo and BeWo cells to DON, inflammatory cytokines, and PM2.5, focusing on inflammatory cytokine production, barrier integrity, and hormone secretion, with the aim of gaining insight into how environmental exposures may impair placental function and fetal development.

## 2. Results

### 2.1. Exposure to DON, CC and PM Did Not Lead to Cytotoxicity in HTR-8/SVneo and BeWo Cells

**Figure 1:**
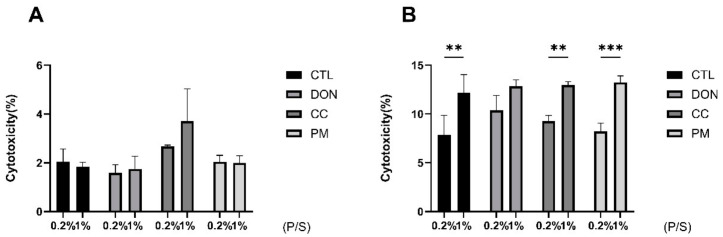
Lactate dehydrogenase (LDH) release in (**A**) HTR-8/SVneo and (**B**) BeWo cells after 24 h incubation with deoxynivalenol (DON), cytokine cocktail (CC), and particulate matter (PM). Cytotoxicity was assessed using the LDH assay. Data are presented as mean ± SEM from three independent experiments, each performed in triplicate. Statistical significance compared to control (CTL) is indicated as follows: ** *p* < 0.01, *** *p* < 0.001.

Upon evaluation of the cytotoxic effects of DON, CC and PM, lactate dehydrogenase (LDH) release was observed to be below 15% in control samples as well as those exposed to the three chosen stimuli. The stimuli were therefore considered non-toxic at the concentrations used in these experiments. Although LDH release by BeWo cells cultured in the presence of 1% penicillin/streptomycin was significantly higher when compared to those cultured in the presence of 0.2% penicillin/streptomycin (Figure 1B), they did not exceed the 15% LDH release and considered non-toxic.

### 2.2. HTR-8/SVneo and BeWo Cells Released IL-6 upon Exposure to DON and CC, but Only HTR-8/SVneo Cells Are Capable of IL-8 Production

**Figure 2:**
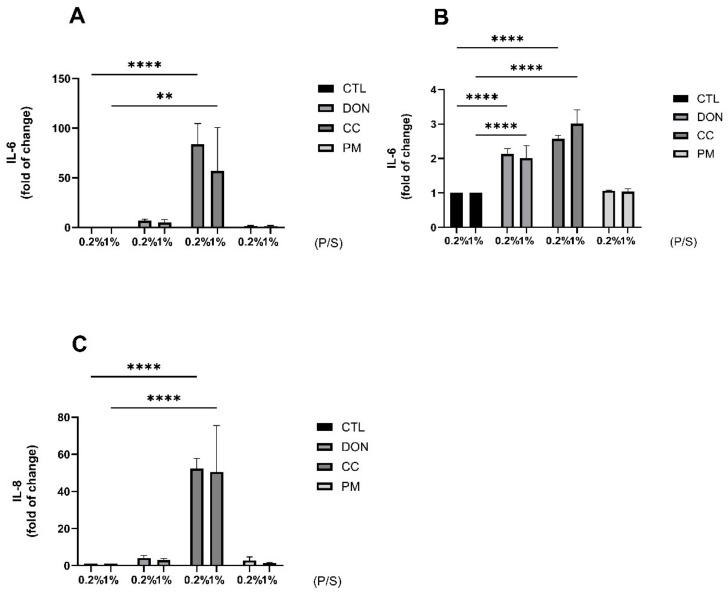
Cytokine secretion by HTR-8/SVneo and BeWo cells after 24 h incubation with deoxynivalenol (DON), cytokine cocktail (CC), and particulate matter (PM). IL-6 secretion was significantly increased only after CC exposure in HTR-8/SVneo cells (Panel (**A**)). In BeWo cells (Panel (**B**)), IL-6 secretion was significantly increased following exposure to DON and CC, whereas PM had no significant effect. In contrast, IL-8 secretion was detectable only in HTR-8/SVneo cells (Panel (**C**)). Data are presented as mean ± SEM from three independent experiments, each performed in triplicate. Statistical significance compared to control (CTL) is indicated as follows: ** *p* < 0.01, **** *p* < 0.0001.

Exposure to DON, CC, and PM resulted in different levels of secretion of IL-6 and IL-8 in HTR-8/SVneo and BeWo cell culture supernatants. In HTR-8/SVneo cells (Figure 2A), exposure to CC significantly increased IL-6 secretion in cell culture supernatants at both concentrations of P/S, compared to their respective controls. In contrast, exposure to DON or PM2.5 did not affect IL-6 secretion at either P/S concentration in HTR-8/SVneo cells.

In BeWo cells (Figure 2B), DON exposure led to a significantly higher secretion of IL-6 at both concentrations of P/S (*p* < 0.0001). Exposure to CC also significantly elevated IL-6 production at both P/S concentrations (*p* < 0.0001). In contrast, exposure to PM2.5 did not affect IL-6 levels at either P/S concentration.

For IL-8 production by HTR-8/SVneo cells (Figure 2C), only CC treatment significantly elevated IL-8 production at both 0.2% and 1% P/S concentrations (*p* < 0.0001), while DON and PM treatments did not show an effect on IL-8 levels. 

### 2.3. Differential Effects of DON and CC on hCG Hormone Production in HTR-8/SV Neo Cells and BeWO Cells

**Figure 3:**
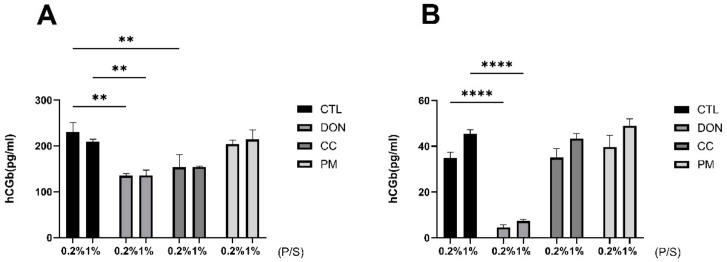
Effects of deoxynivalenol (DON), cytokine cocktail (CC), and particulate matter (PM) exposure on human chorionic gonadotropin (hCG) secretion in HTR-8/SVneo (**A**) and BeWo (**B**) cells. HTR-8/SVneo and BeWo cells were incubated for 24 h with medium (CTL) or with DON, CC, or PM at concentrations of 0.2% and 1% p/s, and hCG secretion was measured. Data are expressed as the mean ± SEM of three independent experiments, each performed in triplicate. Statistical significance compared to control (CTL) is indicated as follows: ** *p* < 0.0001, **** *p* < 0.0001.

Human chorionic gonadotropin (hCG) hormone production by HTR-8/SVneo and BeWo cells was assessed after 24 h exposure to DON, CC, and PM (Figure 3). When comparing control BeWo cells to control HTR-8/SVneo cells, the latter produce larger amounts of hCG (Figure 3A,B).

In HTR-8/SVneo cells (Figure 3A), DON exposure led to a reduction in hCG production compared to the control group (*p* < 0.01). CC exposure significantly decreased hCG by production HTR-8/SVneo cells, but this effect was only observed when cells had been cultured in cell culture medium containing 0.2% P/S (*p* < 0.01) and not 1% P/S. In BeWO cells, only exposure to DON significantly decreased hCG production (Figure 3B) at both concentrations of P/S (*p* < 0.0001). In contrast, no such effects were observed after BeWo cells were exposed to CC or PM treatment. No significant changes in hCG levels were observed after exposure of HTR-8/SVneo cells to PM2.5.

### 2.4. Alterations in mRNA Expression of Junctional Proteins in HTR-8/SVneo and BeWo Cells After Exposure to DON, CC or PM

**Figure 4:**
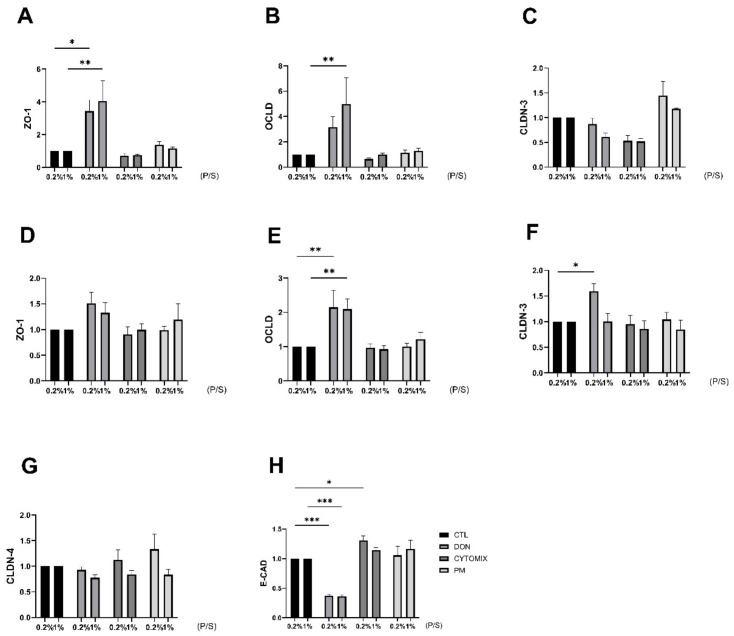
Effects of deoxynivalenol (DON), cytokine cocktail (CC), and particulate matter (PM) exposure on mRNA levels of junctional proteins in HTR-8/SVneo (**A**–**C**) and BeWo (**D**–**H**) cells. The mRNA expression of zonula occludens protein-1 (ZO-1), occluding (OCLD), claudin (CLDN-3 and 4) and E-cadherin (E-CAD) in HTR-8/SVneo and BeWo cells after incubated for 24 h with medium (CTL) or with DON, CC, or PM at concentrations of 0.2% and 1% p/s. Data are expressed as the mean ± SEM of three independent experiments, each performed in triplicate. Y-axis values represent relative mRNA expression calculated using the 2^−ΔΔCt^ method, normalised to β-actin. Statistical significance compared to control (CTL) is indicated as follows: * *p* < 0.05, ** *p* < 0.01, *** *p* < 0.001.

To further examine the effects of environmental and inflammatory stimuli on placental cells, we assessed the mRNA expression levels of five junctional proteins: zonula occludens protein-1 (ZO-1) (Figure 4A,D), occludin (OCLD) (Figure 4B,E), claudin (CLDN)-3 (Figure 4C,F), and CLDN-4 (Figure 4G), and E-cadherin (Figure 4H). In our experiments, CLDN4 and E-cadherin were not expressed at detectable levels in HTR-8/SVneo cells.

Compared to unexposed control HTR-8/SVneo cells, mRNA expression of ZO-1 (Figure 3A) and OCLD (Figure 3B) was significantly higher in cells after DON-exposure, whereas mRNA expression of CLDN-3 was unaffected (Figure 4C). For mRNA expression of OCLD this effect was dependent on the concentration of P/S; only HTR-8/SVneo cells cultured in the presence of 1% P/S were significantly affected after DON-exposure.

In BeWo cells, DON exposure led to a significantly higher mRNA expression of OCLD (Figure 4E) and CLDN-3 (Figure 4F) compared to unexposed control cells, while a significantly lower mRNA expression of E-CAD was observed (Figure 4H). For mRNA expression of CLDN-3 this effect was dependent on the concentration of P/S; only BeWo cells cultured in the presence of 0.2% P/S were significantly affected after DON-exposure. No significant differences were observed in ZO-1 or CLDN-4 mRNA expressions.

No significant changes in mRNA expression were found when either cell type was exposed to PM2.5 (Figure 3), and mRNA expression levels were unaffected in HTR-8/SVneo cells exposed to CC. In BeWo cells, CC-exposure affected only E-CAD mRNA expression which was found to be significantly higher compared to that of unexposed control cells, but only when cells had been cultured in 0.2% P/S.

### 2.5. Effects of DON, CC and PM on Protein Expression of Junctional Proteins

**Figure 5:**
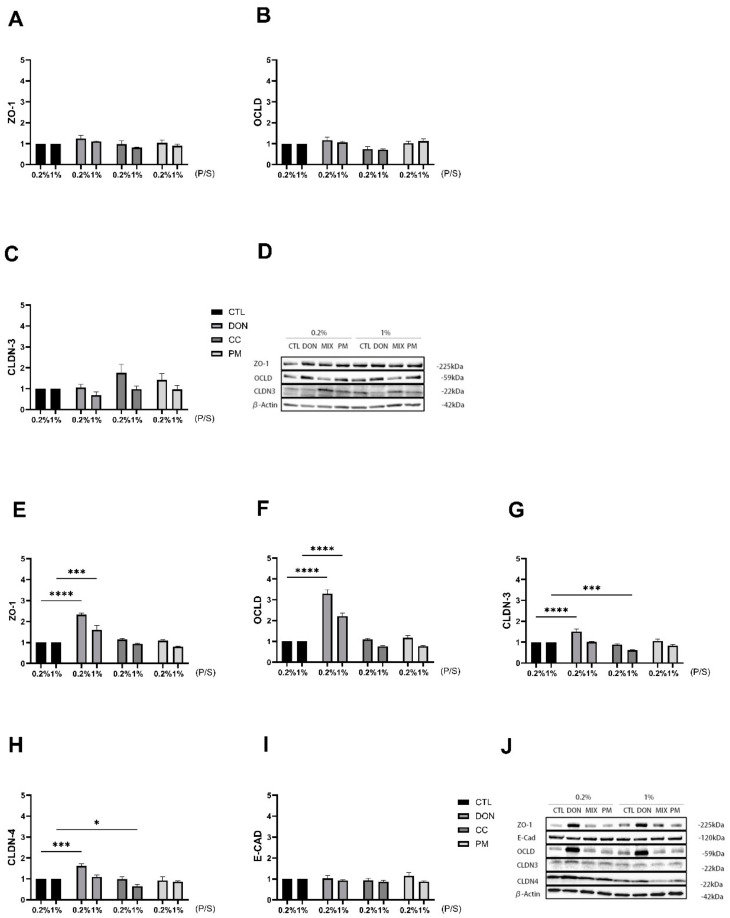
Effects of deoxynivalenol (DON), cytokine cocktail (CC), and particulate matter (PM) exposure on protein expression levels of junctional proteins in HTR-8/SVneo (**A**–**D**) and BeWo (**E**–**H**) cells. The protein expression levels of occludin (OCLD), zonula occludens protein-1 (ZO-1), claudin (CLDN)-3 and 4, and E-cadherin (E-CAD) were quantified in HTR-8/SVneo (**A**–**D**) and BeWo cells (**E**–**I**) after 24 h of exposure to medium (CTL), DON, CC, and PM. Representative Western blot images are shown in (**J**). Data are expressed as the mean ± SEM of three independent experiments, each performed in triplicate. Y-axis values represent relative protein expression determined by densitometric analysis of Western blot bands, normalised to β-actin as the loading control. Statistical significance compared to control (CTL) is indicated as follows: * *p* < 0.05, *** *p* < 0.001, **** *p* < 0.0001.

To further evaluate the effects of a 24 h exposure to DON, CC, or PM on HTR-8/SVneo and BeWo cells, we analyzed protein expression levels of the junctional proteins using Western blot analysis (Figure 5). Corresponding to the results of the qPCR-analyses, protein expression of E-cadherin and CLDN4 could not be detected in HTR-8/SVneo cells.

#### 2.5.1. Expression of Junctional Proteins After DON Exposure

In contrast to the mRNA expression in HTR-8/SVneo cells, DON exposure did not induce any significant changes in the protein expression of ZO-1, OCLD. No effects were observed in protein expression of CLDN3 under either 1% or 0.2% P/S conditions, compared to the control (Figure 4A–D).

In BeWo cells, in agreement with the mRNA expression, DON significantly increased the protein levels of ZO-1 (Figure 4E) and OCLD (Figure 4F) under both 1% and 0.2% P/S conditions (*p* < 0.001 or *p* < 0.0001). CLDN3 (Figure 4G) and CLDN4 (Figure 5H) were also significantly upregulated under 0.2% P/S. In contrast to mRNA expression, no significant change was observed in E-CAD expression (Figure 5I). These changes were consistent with the representative Western blot bands (Figure 5J).

#### 2.5.2. Expression of Junctional Proteins After CC Exposure

In HTR-8/SVneo cells, CC exposure did not affect the protein expression of ZO-1, OCLD, or CLDN3 under either P/S condition (Figure 5A–D), which is consistent with the mRNA expression results. In BeWo cells, CC significantly downregulated CLDN3 (Figure 5G) and CLDN4 (Figure 5H) protein levels under 1% P/S, despite no significant changes at the mRNA level. No significant changes were observed in ZO-1, OCLD, or E-CAD protein levels (Figure 5E–I), which is in line with the mRNA findings, except for E-CAD, where increased mRNA expression did not result in higher protein expression.

#### 2.5.3. Expression of Junctional Proteins After PM Exposure

In HTR-8/SVneo and BeWo cells, PM2.5 exposure did not result in significant changes in the protein expression of ZO-1, OCLD, CLDN3, CLDN4, or E-CAD under either P/S condition (Figure 5A–J). This aligns with the mRNA expression data, which also showed no significant alterations in any of the five junctional proteins (Figure 3). A summary of these findings is presented in Table 1.

## 3. Discussion

Maternal factors, including those derived from the outside environment, can disrupt the intricate structure of the placenta affecting its function and thereby exerting influence on fetal growth. Such changes may have enduring implications for long-term health outcomes. To gain further understanding of the potential impact of such maternal factors on placental immune response, structure, and barrier proteins, we investigated the responses of two placental cell lines, BeWo and HTR-8/SVneo cells upon exposure to DON, CC, and PM. Results from preliminary experiments in our laboratory suggest that HTR-8/SVneo cells and BeWo cells were less responsive to stimuli when antibiotics were present in the cell culture medium. We therefore compared the impact of exposure to these stimuli for cells cultured in the presence of conventional concentrations of antibiotics (1% P/S) and those cultured in medium containing a low concentration of antibiotics (0.2% P/S).

### 3.1. Impact of DON on Placental Cell Function: Inflammation, Hormonal Disruption, and Altered Tight Junction Integrity

Mycotoxin deoxynivalenol (DON) is commonly detected in agricultural products and human foodstuffs, making exposure unavoidable [19,20]. Pregnant women can be exposed to DON at levels that exceed tolerable daily intake [9,21] and in animal studies, DON has been demonstrated to crosses the placenta, posing potential risks to fetal development and maternal health [10,22,23,24]. In line with our earlier observations [25], exposure to DON at a concentration of 4 µM did not significantly impact cell viability in BeWo and HTR-8/SVneo cells in the current study. Furthermore, a significant increase in the secretion of IL-6 could be observed in both cell lines, and IL-8 secretion was increased in HTR-8/SVneo cells. To our current knowledge, we are the first to report that DON can induce the production of IL-6 and IL-8 in HTR8/SVneo cells. Elevated IL-6 and IL-8 levels are linked to adverse pregnancy outcomes, such as placental dysfunction and fetal growth restriction, primarily through their roles in inflammatory responses [26,27,28,29,30,31]. Exposure to DON significantly suppressed human chorionic gonadotropin (hCG) production in both BeWo and HTR-8/SVneo cell lines. Previous studies have established that disruptions in human chorionic gonadotropin (hCG) production are associated with adverse pregnancy outcomes, such as miscarriage and preeclampsia. DON exposure was shown to decrease hCG secretion dose-dependently in BeWo cells, impairing placental hormone production. The higher basal hCG levels in HTR-8/SVneo cells likely reflect lineage-specific traits: HTR-8/SVneo cells derive from extravillous trophoblasts, which exhibit low basal hCG expression. In contrast, BeWo cells require forskolin-induced syncytialization to markedly increase hCG secretion. Thus, the elevated hCG in untreated HTR-8/SVneo cultures is consistent with their EVT-like phenotype [32]. Similarly, zearalenone, another mycotoxin, was found to disrupt hCG production and negatively affect pregnancy outcomes.

In the present study, our data show that DON exposure in BeWo cells led to the upregulation of mRNA expression of ZO-1, OCLD, and CLDN-3, while decreasing E-cadherin mRNA expression. At the protein level, both ZO-1 and OCLD showed increased expression, which may suggest a compensatory response to stress. Interestingly, CLDN-4 protein levels were elevated without corresponding mRNA changes, indicating potential post-transcriptional regulation. E-cadherin protein levels did not decrease significantly under the conditions tested. These observations differ from findings by Seyed Toutounchi et al. [25] and De Walle et al. [33] who observed upregulated mRNA expression of ZO-1 and OCLD but found no change in CLDN-3 and CLDN-4 levels, while E-cadherin mRNA was downregulated in BeWo cells exposed to DON. Seyed Toutounchi et al. [25] reported reductions in ZO-1, CLDNs, and E-cadherin protein levels with no significant change in OCLD protein expression. Similarly, De Walle et al. [33] found that DON exposure in Caco-2 cells increased the transcript levels of claudin-4 and occludin more than threefold, but protein levels decreased. These discrepancies might be reflecting biological variability inherent in vitro systems, such as passage-dependent changes, subtle fluctuations in culture conditions, or differences in cellular metabolic states at the time of treatment. While the exact cause remains unclear, these results underscore the importance of experimental replication and cautious interpretation of subtle quantitative differences. To our knowledge, no previous studies have examined the effect of DON on HTR-8/SVneo cells specifically and although there is clear variation in the outcomes on adhesion tight junction proteins in BeWO cells, results suggest that environmental triggers may alter the junctional organisation of trophoblast-like cells. However, it should be noted that changes in junctional marker expression represent indirect indicators of barrier function; functional assays such as trans-epithelial electrical resistance (TEER) measurements or paracellular permeability assays would be required to directly confirm effects on barrier integrity. These observations nonetheless underscore the importance of investigating the potential detrimental effects of DON on placental cell function. These differences may be attributed to the mixed epithelial and mesenchymal-like cell population in HTR-8/SVneo cells, which could attenuate the cellular response to DON compared to the more uniform BeWo cells. Additionally, the absence of detectable E-cadherin and CLDN-4 in HTR-8/SVneo cells, in contrast to the measurable E-cadherin expression in BeWo cells, may be attributed to their mesenchymal-like nature-characterized by reduced epithelial marker expression and increased cellular motility. These differences may reflect the complex nature of trophoblast responses to toxins like DON and underscore the importance of selecting the appropriate cell models for studying placental toxicity.

### 3.2. Impact of CC on Placental Cell Function: Inflammation, Hormonal Disruption, and Altered Tight Junction Integrity

In this study, exposure to a cocktail of inflammatory cytokines (TNF-α, IFN-γ, and IL-1β) significantly increased the secretion of IL-6 in both BeWo and HTR-8/SVneo cells, simulating the inflammatory environment that the placenta might encounter due to maternal inflammation. This is in line with earlier observations by Fujisawa, who reported that TNF-α and IL-1β elevate IL-6 levels in BeWo cells [34].

Our research builds on these findings by specifically examining the differential response of BeWo and HTR-8/SVneo cells to inflammatory cytokines. Strikingly, in HTR-8/SVneo cells, exposure to CC significantly increased IL-6 and IL-8 secretion, 100-fold and 40-fold higher than the non-treated group, respectively. This suggests that extravillous trophoblasts (EVTs, represented by HTR-8/SVneo cells) are particularly susceptible to inflammatory stimuli, leading to substantial cytokine production. This response highlights the significant impact of inflammation on placental cells, emphasizing the potential relevance of modulating inflammatory responses during pregnancy to mitigate adverse outcomes. However, findings from in vitro cell cultures should be interpreted with caution, as they do not fully replicate the complexity of human placental tissue. While elevated IL-6 levels in human placental tissue have been linked to impaired endothelial cell homeostasis and placental vascularization, leading to conditions such as intrauterine growth restriction (IUGR) and preeclampsia (PE), these outcomes highlight the need for further validation to bridge the gap between cell line studies and human pathology.

Differential responses of BeWo and HTR-8/SVneo cells to the inflammatory cytokine cocktail (CC) were observed, particularly in the regulation of junctional proteins, which are critical for maintaining placental barrier integrity under inflammatory conditions. In BeWo cells, CC exposure resulted in the upregulation of E-cadherin (E-cad) mRNA levels without corresponding protein changes, suggesting post-transcriptional regulation. CLDN3 and CLDN4 protein levels were significantly reduced, suggesting that tight junction proteins may be vulnerabl to inflammatory stimulation. These changes in junctional protein expression may be indicative of potential alterations in junctional organisation, though functional validation would be required to confirm effects on barrier integrity. This aligns with previous studies in Caco-2 cells [35] demonstrated that decreasing ZO-1 and occludin levels after cytokine exposure. In contrast, HTR-8/SVneo cells showed no significant changes in the mRNA or protein levels of ZO-1, OCLD, or CLDN3, which may reflect intrinsic differences in cell population composition and tight junction regulation compared to BeWo cells. This may support the invasive function and migratory capabilities of extravillous trophoblasts during placentation [36,37,38,39]. These results highlight distinct, cell-type-specific regulatory responses of placental cells to inflammation conditions.

### 3.3. Effect of PM Exposure on Inflammatory Markers and hCG Levels in Placental Cells: Insights into Duration and Concentration Effects

There is a recognized association between PM pollution during pregnancy and complications such as preterm birth and preeclampsia, with placental dysfunction potentially contributing to these outcomes. Studies have detected particulate matter within placental cells, suggesting the placenta may be directly impacted by air pollution. However, understanding the effects of PM on placental function remains challenging, as data from various studies show inconsistent results, highlighting the need for further research before drawing definitive conclusions.

Our findings indicate that PM exposure did not significantly affect IL-6, IL-8, hCG levels, or tight junction and adherens proteins in BeWo and HTR-8/SVneo cells. This contrasts with studies such as those by Familari et al. (2019), who observed increased IL-6 and IL-8 levels upon PM exposure and Nääv et al. (2020), who reported decreased hCG and elevated IL-6 after 48 h of exposure on HTR-8/SVneo cells [40,41]. These discrepancies may be attributed to differences in experimental conditions, such as the PM concentration (5 or versus 10 µg/mL), exposure duration (48 h versus 24 h in the current study). Qin et al. (2017) also highlighted the importance of exposure duration and concentration, as they found that PM2.5 induced cell cycle arrest and inhibited migration and invasion in HTR-8/SVneo cells after longer (48h) exposure [42]. This is consistent with the possibility that the absence of significant changes in our 24 h exposure experiments may be due, at least in part, to insufficient exposure duration to elicit comparable functional alterations in trophoblast cells.

Although no significant changes were detected in inflammatory markers or hCG levels, our results revealed cellular alterations upon PM exposure, indicating that PM can still modulate placental cell responses. These observations warrant further investigation into the underlying mechanisms and their implications for placental function and fetal health.

## 4. Materials and Methods

### 4.1. Cell Culture

The BeWo cell line (ATCC-CCL-98, Rockville, MD, USA), derived from human placental choriocarcinoma, is a well-established and widely used in vitro model for studying syncytiotrophoblast function, including hormone secretion and placental transport [1]. Despite their cancer origin, BeWo cells express key trophoblast markers and produce hCG, making them a standard reference model in placental research, was cultured at 37 °C with 5% CO2 in F-12K medium (Gibco, Thermo Fisher Scientific, Wilmington, DE, USA). The culture medium was supplemented with 10% fetal calf serum (Perbio Science, Brebières, France) and 1% penicillin/streptomycin.

The HTR-8/SVneo cell line (ATCC-CRL-3271, Rockville, MD, USA) was cultured in a T-75 flask using RPMI 1640 medium enriched with L-glutamine and sodium bicarbonate. The medium was supplemented with 10% fetal calf serum, 1% penicillin/streptomycin (P/S) solution, 5 mL of 1 M HEPES (Gibco, Thermo Fisher Scientific, USA), and 5 mL of 100 mM sodium pyruvate (Gibco, Thermo Fisher Scientific, USA).

For the experiments, both HTR-8/SVneo and BeWo cells were seeded in 24-well plates (Falcon, VWR International, Strasbourg, France) at densities of 30,000 cells per well with either 0.2% or 1% penicillin/streptomycin. Once the cells reached 80% confluency, they were exposed to 4 µM Deoxynivalenol (DON, Sigma-Aldrich, St. Louis, MO, USA), cytokine cocktail (CC, consisting of TNFα, IL-1β, and IFNγ, Invitrogen, Thermo Fisher Scientific, Wilmington, DE, USA), and 10 µg/mL particulate matter (PM, NIST2786, Sigma-Aldrich, St. Louis, MO, USA), either 0.2% or 1% P/S, for 24 h. Two concentrations of P/S were compared as a methodological validation step, based on preliminary unpublished observations from our laboratory suggesting that standard antibiotic concentrations may modulate cellular responsiveness to certain stimuli. The concentration for DON was based on previous studies from our laboratory [25]. The concentrations of cytokines in the CC (TNF-α, IL-1β, and IFN-γ, each at 10 ng/mL) were selected based on published studies demonstrating effective inflammatory activation in trophoblast cell lines [34]. The PM2.5 concentration (10 µg/mL) was selected based on published studies using similar urban particulate matter preparations in trophoblast models [41,43]. After exposure, the medium was collected for subsequent analyses of cytotoxicity, hormone production, and cytokine release, while cell lysates were collected for Western blot and qPCR analysis.

### 4.2. Cell Cytotoxicity Assay

To assess cytotoxicity of DON, CC and PM on HTR-8/SVneo and BeWo, 50 µL of supernatant of each well was transferred to a new 96-well plate and the content of lactate dehydrogenase was determined by using the CytoTox 96^®^ Non-Radioactive CytoToxicity Assay Kit (Promega Corporation, Madison, WI, USA) according to the manufacturer’s instructions. Cytotoxicity (%) was calculated using the formula: Cytotoxicity (%) = (E/M) × 100%, wherein E represents the experimental LDH release and M denotes the maximal LDH release, ascertained through cellular incubation with a standardized lysis solution, as provided in the assay kit.

### 4.3. Detection of IL-6, IL-8 and Human Chorionic Gonadotropin in Cell Culture Supernatants

IL-6, IL-8 and hCG were measured in cell culture supernatants of HTR-8/SVneo and BeWo cells using commercially available human IL-6 and IL-8 enzyme-linked immunosorbent assay (ELISA) kits (Invitrogen, San Diego, CA, USA) and the human CG ELISA kit (R&D Systems, Minneapolis, MN, USA) following the guidelines provided by the manufacturer. In short, the supernatants were collected and centrifuged to remove cell debris. The clear supernatants were then added to the ELISA plates pre-coated with antibodies specific to either IL-6, IL-8 or hCG. After an incubation period to allow binding, a detection antibody conjugated to an enzyme was added. Following further incubation and washing steps to remove unbound components, a substrate solution was added to the wells. The enzymatic reaction produced a color change proportional to the concentration of IL-6, IL-8 or hCG present in the samples. Absorbance was measured at 450 nm using a microplate reader (Bio-Rad, Hercules, CA, USA), and the cytokine concentrations were quantified by comparing the absorbance values to a standard curve generated from known concentrations of IL-6 or IL-8.

### 4.4. RNA Extraction and Quantitative qPCR

RNA extraction was performed using the Qiagen RNeasy Mini Kit (Qiagen, Crawley, UK). Both HTR-8/SVneo and BeWo cells were seeded in 24-well plates as described above and exposed to different stimuli for a duration of 24 h. Hereafter, cells were lysed using RNA lysis buffer containing dithiothreitol (DTT, 50 mM, Thermo Fisher, Wilmington, DE, USA), and isolation of total RNA was carried out using spin columns in accordance with the manufacturer’s guidelines. The NanoDrop ND-1000 Spectrophotometer (Thermo Fisher Scientific, Wilmington, DE, USA) was used to assess the quantity and quality of total RNA in each sample. To generate cDNA, the iScript cDNA Synthesis kit (Bio-Rad Laboratories, Hercules, CA, USA) was utilized on the total RNA samples. For qPCR, the reaction mixture was prepared by mixing the samples with specific forward and reverse primers 19, along with iQSYBR Green Supermix (Bio-Rad Laboratories, Hercules, CA, USA). The primer sequences used for qRT-PCR analysis are listed in Table 2. The reaction mixture was added to the samples, and amplification was carried out using the CFX96 Touch™ Real-Time PCR Detection System (Bio-Rad Laboratories, Hercules, CA, USA), following the instructions provided by the manufacturer. Relative levels of mRNA expression were determined by normalizing the expression of the genes of interest to the expression level of the β-actin reference gene.

### 4.5. Western Blot Analysis

Western blot analyses were used to assess protein expression levels of tight junction proteins in HTR-8/SVneo and BeWo cells. Cell lysis and harvesting were performed using RIPA Lysis and Extraction Buffer (Thermo Scientific, Rockford, IL, USA) supplemented with protease inhibitors (one tablet in 10 mL RIPA buffer) according to the manufacturer’s instructions (cOmplete, Roche Applied Science, Penzberg, Germany). The total protein concentration was determined using a BCA protein assay kit (23225, Thermo Scientific, Rockford, IL, USA). Equivalent amounts of protein from heat-denatured samples were separated via electrophoresis using a Stain-free Criterion™ Gel (4–20% Tris-HCl, Bio-Rad Laboratories, Hercules, CA, USA) and transferred to Trans-Blot Turbo Midi PVDF Transfer Packs (Bio-Rad Laboratories, Hercules, CA, USA) using the Trans-Blot Turbo Transfer System (Bio-Rad Laboratories, Hercules, CA, USA). Subsequently, the membranes were blocked with a blocking buffer (0.05% (*v*/*v*) Tween-20 in PBS (PBST) and 5% (*w*/*v*) milk (Santa Cruz Biotechnology, Santa Cruz, CA, USA) for 1 h at room temperature. Primary antibodies, diluted in blocking buffer according to the manufacturer’s instructions, were then incubated with the membranes overnight at 4 °C. Following three washes with PBST, the membranes were incubated with anti-rabbit (1:3000, P0448 Dako, Glostrup, Denmark) or anti-mouse (1:3000, P0447, Dako, Glostrup, Denmark) secondary antibodies for 1 h at room temperature. After washing with PBST, the blots were exposed to Clarity Western ECL reagents mix (Bio-Rad, Hercules, CA, USA) and imaged using the ChemiDoc™ MP+ System (Bio-Rad Laboratories, Hercules, CA, USA). The results obtained were analyzed using Image J software (version 1.52, National Institutes of Health, Bethesda, MD, USA). The primary antibodies used in this study included CLDN-3 (1:500) and CLDN-4 (1:1000), ZO-1 (1:1000, 341700, 329400, and 402200, Invitrogen, Carlsbad, CA, USA), OCLD (1:500, AB31721, Abcam, Cambridge, UK), and E-cadherin (1:1000, 610182, eBioscience, San Diego, CA, USA).

### 4.6. Statistical Analysis

Data were analyzed using GraphPad Prism version 10.0 (GraphPad Software, San Diego, CA, USA). The results of three independent experiments are presented as mean ± standard error of the mean (SEM). Differences among groups were assessed using two-way analysis of variance (ANOVA). Post hoc comparisons of each treatment group (DON, CC, and PM) versus the corresponding control group were performed using Dunnett’s test, which is specifically designed for comparing multiple treatment groups to a single control while controlling for family-wise error rate. A *p*-value of less than 0.05 was considered statistically significant.

## 5. Conclusions

Our study reveals the differential effects of deoxynivalenol (DON), inflammatory cytokine cocktail (CC), and particulate matter (PM) on cytokine secretion, human chorionic gonadotropin (hCG) production, and the mRNA and protein expression of junctional markers in BeWo and HTR-8/SVneo placental cell lines. To our knowledge, this is the first report showing that DON significantly increases IL-6 and IL-8 secretion in HTR-8/SVneo cells. While DON suppresses hCG production in both cell lines, CC does not affect hCG levels, highlighting the resilience of hCG production under inflammatory conditions. Our study also found that PM exposure did not significantly affect IL-6, IL-8, or hCG levels in either cell line. At the molecular level, DON and CC altered the expression of junctional markers in a cell-line-dependent manner, suggesting potential alterations in trophoblast junctional organisation; however, functional assays (e.g., TEER or permeability measurements) would be required to directly confirm effects on barrier integrity. The majority of cellular responses were consistent across both P/S concentrations (0.2% and 1%), indicating that the observed responses are robust to antibiotic concentration within the tested range, and that the observed effects are attributable to the stimuli themselves rather than to the antibiotic concentration used in trophoblast cell culture studies. Together, these results demonstrate that HTR-8/SVneo and BeWo cells exhibit differential sensitivity to exposome-related stimuli, emphasising the importance of cell model selection in in vitro placental toxicology research. Given that exposure to such environmental factors during pregnancy is often unavoidable, these findings warrant further investigation of the underlying mechanisms and their implications for placental function and fetal health.

## Figures and Tables

**Table 1:** Summarized overview of the effects of deoxynivalenol (DON), cytokine cocktail (CC), and particulate matter (PM) on the mRNA and protein expression levels of occludin (OCLD), zonula occludens protein-1 (ZO-1), claudin (CLDN)-3 and -4, and E-cadherin (E-cad) in BeWo and HTR-8/SVneo cells.

TJ or AJ	BeWo		HTR-8/SVneo	
	mRNA	Protein	mRNA	Protein
**DON**				
ZO-1	–	↑	↑	–
OCLD	↑	↑	↑	–
CLDN-3	↑	↑	↑	–
CLDN-4	–	↑	*	*
E-cad	↓	–	*	*
**CC**				
ZO-1	–	–	↑	–
OCLD	–	–	↑	–
CLDN-3	–	↓	↑	–
CLDN-4	–	↓	*	*
E-cad	↑	–	*	*
**PM**				
ZO-1	–	–	–	–
OCLD	–	–	–	–
CLDN-3	–	–	–	–
CLDN-4	–	–	*	*
E-cad	–	–	*	*

↑, significantly increased; ↓, significantly decreased; –, no significant change; *, not determined because CLDN-4 and E-cadherin were not detectable in HTR-8/SVneo cells.

**Table 2:** Primer sequences used for qRT-PCR analysis. AT: annealing temperature (°C).

Genes	Primer Sequence (5′–3′)		References	AT
	Forward	Reverse		
*ACTB*	CTGGAACGGTGAAGGTGACA	AAGGGACTTCCTGTAACAATGCA	NM-001101	63
*CLDN3*	CTGCTCTGCTGCTCGTGTC	CGTAGTCCTTGCGGTCGTAG	NM-001306	63
*CLDN4*	GTCTGCCTGCATCTCCTCTGT	CCTCTAAACCCGTCCATCCA	NM-001305	62.5
*CDH1*	TGGACCGAGAGAGTTTCCCT	CCCTTGTACGTGGTGGGATT	BC-144283.1	60
*OCLN*	TTGGATAAAGAATTGGATGACT	ACTGCTTGCAATGATTCTTCT	NM-002538	57
*TJP1*	GAATGATGGTTGGTATGGTGCG	TCAGAAGTGTGTCTACTGTCCG	NT-010194.17	55.8

## Data Availability

The original contributions presented in this study are included in the article. Further inquiries can be directed to the corresponding author.

## Abbreviations

The following abbreviations are used in this manuscript:

DON	Deoxynivalenol
PM	Particulate Matter
CC	cytokine cocktail
IL-6	Interleukin-6
IL-8	Interleukin-8
ELISA	enzyme-linked immunosorbent assay
TNFa	Tumor Necrosis Factor-alpha
IL-1b	Interleukin-1 beta
IFNy	Interferon-gamma
hCG	human chorionic gonadotropin
